# Contextual factors affecting the implementation of drug checking for harm reduction: a scoping literature review from a North American perspective

**DOI:** 10.1186/s12954-023-00856-0

**Published:** 2023-09-04

**Authors:** Chloe Grace Rose, Victoria Kulbokas, Emir Carkovic, Todd A. Lee, A. Simon Pickard

**Affiliations:** Department of Pharmacy Systems, Outcomes, and Policy, University of Illinois Chicago, 833 S Wood St, Chicago, IL 60612 USA

**Keywords:** Drug checking, Drug checking services, Drug checking technologies, Harm reduction

## Abstract

**Background:**

The opioid epidemic continues to be a significant cause of morbidity and mortality in the US. In 2020, 83% of opioid-related overdose deaths were due to synthetic opioids, such as fentanyl. Drug checking services have been widely implemented as a harm reduction intervention to facilitate the identification of substances in a drug sample. There is a need to inform decision-making on drug checking technologies and service implementation. This research aims to outline contextual considerations for the implementation of a drug checking service.

**Methods:**

A scoping review was conducted using a structured search strategy in PubMed and EMBASE. Articles were independently screened by two reviewers, and included if they were primary literature and reported on an actionable consideration(s) for drug checking services. Data elements were extracted using a standardized form, and included study design, study population, drug checking technology utilized or discussed, and main findings.

**Results:**

Twenty-nine articles were selected for inclusion, and four primary areas of consideration were identified: drug checking technologies, venue of a drug checking service, legality, and privacy. Technological considerations include the need for highly accurate, quantitative results which appeal to both populations of people with drug use disorder and recreational users. Accessibility of services was identified as an important factor that may be impacted by the location, integration with other services, how the service is provided (mobile vs. fixed), and the hours of operation. Maintaining plausible deniability and building trust were seen as important facilitators to service use and engagement. Issues surrounding legality were the most frequently cited barrier by patrons, including fear of criminalization, policing, and surveillance. Patrons and stakeholders identified a need for supportive policies that offer protections. Maintaining anonymity for patrons is crucial to addressing privacy-related barriers.

**Conclusion:**

This review highlights the need to understand the local population and climate for drug checking to implement a drug checking service successfully. Common themes identified in the literature included considerations related to the choice of technology, the type of venue, and the impact of legality and privacy. We intend to utilize these considerations in future research to help guide discussions with US-based stakeholders.

**Supplementary Information:**

The online version contains supplementary material available at 10.1186/s12954-023-00856-0.

## Introduction

Since 1999, opioid-related overdose deaths in the USA have been on the rise [1]. In 2020, deaths reached 21.4 per 100,000, with the majority (83%) due to synthetic opioids, such as fentanyl. Similarly, this dangerous trend is also seen in Illinois, with the rate of fatal overdoses being 24.0 per 100,000 in 2021 [2]. Overall, 3,013 people died from opioid drug overdoses in 2021, representing a 2.3% increase in opioid-related deaths from 2020 and a 36% increase from 2019, respectively. Some of the evidence-based strategies for preventing opioid overdose include targeted naloxone distribution, academic detailing (a form of one-on-one, evidence-based educational outreach to healthcare providers), syringe service programs (SSPs), and screening for fentanyl in routine clinical toxicology testing; the latter suggested to help facilitate surveillance of the local drug supply and provide early warning of contamination [3]. A full list of abbreviations is provided following the conclusion.

Drug checking services have been implemented widely across Europe and Australia as a harm reduction strategy [4, 5]. Drug checking services are used to identify the composition of a drug sample and provide an opportunity for people to test their drugs prior to or following use. Given the rise in opioid-related deaths and fentanyl adulteration in the illicit drug supply [6], drug checking services may provide service users with greater knowledge and control in preventing opioid-related overdose.

Several technologies have been used for drug checking [7]. Fentanyl test strips (FTS), created to detect fentanyl in urine samples, are a fast and affordable method of drug checking. FTS are highly accurate, and results are produced rapidly within seconds to minutes and are easily read by an operator with minimal training. However, FTS are limited to detecting fentanyl, some fentanyl analogs, and cannot produce quantitative results [8]. In addition to FTS, test strips for other drugs/chemical reagents are available. Another commonly used technology is Fourier-transform infrared spectroscopy (FTIR). FTIR has high discriminatory power and can detect virtually all substances when interfaced with an electronic library. FTIR is more portable than traditional laboratory-based technologies, like mass spectrometry (MS), and may be more suitable in a point-of-care setting. FTIR does not result in destruction of the sample and can produce results within minutes. FTIR has a relatively high detection limit (varies by device) which can restrict it from detecting highly potent drugs such as fentanyl or its analogs. Additionally, it is not an effective technology for identifying new substances. On the other hand, MS machines paired with gas or liquid chromatography (GS-MS, LC–MS) are the gold standard for drug identification. MS offers the highest level of discriminatory ability, can identify virtually any substance by interfacing with a library of compounds, and is capable of identifying new substances. MS technologies are highly sensitive and can detect compounds at ultralow concentrations, unlike FTIR. However, both FTIR and MS require advanced operator knowledge and may be cost-prohibitive.

While many technical specifications inform the appropriateness of a drug checking technology, there may be additional contextual factors and criteria relevant to stakeholders when making decisions about resource allocation concerning drug checking technologies. These criteria can be informed by reviewing the literature, and consulting drug checking service patrons and stakeholders involved in harm reduction. The aim of this research is to review contextual factors to identify drug checking technologies that are most suitable for implementation in a harm reduction service within the context of the opioid epidemic.

## Methods

### Search strategy

We conducted a scoping literature review by identifying potential articles through queries in PubMed and EMBASE. No appropriate MeSH terms were identified in PubMed. Therefore, titles and abstracts were searched using the following strategy: (“drug” or “opioid” or “fentanyl” or “pill” or “substance”) AND “checking”. EMBASE was searched using the candidate term “drug checking.”

### Inclusion and exclusion criteria

Search criteria included unique, full-text articles published through July 10, 2022. Articles were included if they were original primary literature and written in English. In addition, articles were included if they reported on an actionable consideration for a drug checking service, including any factors that could impact decision-making regarding drug checking technologies, or how drug checking services are offered. Articles were excluded if they did not provide actionable results (e.g., reported local drug market trends, or reported behavior change or intent to change as a measure of program effectiveness without a comparator) or described technologies of limited interest due to technical limitations (e.g., colorimetric testing).

Of note, drug checking services are used by both people who use drugs (PWUD) and people who use party drugs (PWUPD). PWUPD may be thought of as social drug users and engage in substances use with drugs such as cocaine, MDMA, and ecstasy [9]. This population differs from systemically vulnerable PWUD in many ways. Compared to PWUPD, PWUD may be of lower socioeconomic status and engage in substance use more regularly with drugs such as heroin or other opioids. This review includes articles on both populations of PWUD and PWUPD.

### Review strategy

During initial screening, article titles and abstracts were reviewed by two independent reviewers (CGR and VK). The remaining articles were retrieved, and full manuscripts were selected by two independent reviewers (CGR and EC) based on the inclusion and exclusion criteria. Reviewers met to discuss and resolve selection discrepancies at each stage. Data elements of interest were then extracted from the selected articles using a standardized form. The standardized form included study citation, source, study design, study population, drug checking technology utilized or discussed, and main findings. All review and data extraction were completed using Covidence (2022) [10].

## Results

**Fig. 1:**
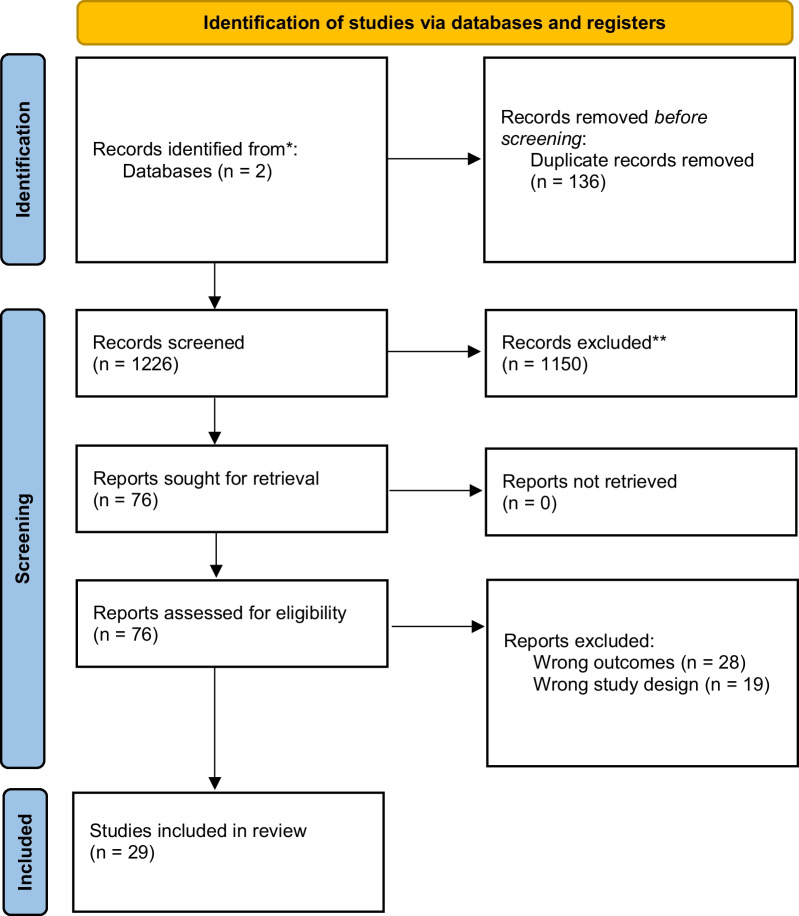
PRISMA flow diagram of article screening and inclusion

A total of 1362 citations were identified by database search and imported for screening. There were 136 duplicate records removed, resulting in 1,226 unique citations. After title and abstract screening, the remaining 76 full texts were evaluated based on inclusion and exclusion criteria. Twenty-nine articles were included in the review (Fig. 1).

Four major themes were identified: considerations pertaining to drug checking technologies, considerations related to the venue of a drug checking service, and concerns related to legality, and privacy (Table 1). First, we summarize results that pertain to drug checking technologies. This includes patron perspectives and needs related to drug checking technologies, and considerations for three types of drug checking technologies discussed in the included articles: FTS, FTIR and FTIR technology combinations, and MS. Next, we summarize findings related to the venue of a drug checking service, including patron perspectives on location and accessibility, considerations for integrating drug checking with other services, and considerations related to drug checking staff (other). Finally, we summarize barriers regarding legality and privacy, and their impact upon service utilization. A list of the included studies and key findings can be viewed in Table 2 (see Additional file 1).

**Table 1:** Summary of identified themes

Theme	Key findings
Drug checking technologies	
Patron perspectives	Wait time, cost, and sample destruction were identified as barriers to service utilization Highly accurate, quantitative results are preferred by patrons
Fentanyl test strips	Fentanyl test strips are seen as highly accepted and easy to use A wide variety of locations were suggested as distribution sites
Fourier-transform infrared spectroscopy (FTIR)	Use of FTIR was commonly reported in the literature, with variable results Benefits include broad range of substances detected, lower cost, and improved portability compared to MS technologies Limitations include a high detection limit, and poorer sensitivity and specificity compared to laboratory-based technologies
Mass spectrometry (MS)	New MS technologies have been employed for harm reduction and may offer improved technological specifications (detection limits, accuracy, etc.) compared to FTIR Limitations include high cost, expertise needed for operation, and physical site requirements
Venue	
Patron perspectives	Patrons emphasized the need for assessable services with convenient locations and hours of operation Mobile services may help facilitate plausible deniability, outreach to rural patrons, and adaptive response to local drug trends by allowing services to be provided in high-need or high-risk areas
Integration of services	Integration of drug checking with other services was reported with mixed results Integration may capitalize on trust and comfortability if patrons already use services at an existing site, and may help bridge patrons to other harm reduction services Integration of services may be a barrier to some patrons who fear stigma associated with a site or its services
Other	Peers or people with lived experience were seen as important for developing trust in patrons Communication of results should be informative on the substances identified and appeal to a wide range of patrons
Legality	Legality was the most commonly reported barrier to service utilization Criminalization was a major concern for both patrons and staff members
Privacy	Patrons are concerned with being identified physically, and through inappropriate use of their information

### Considerations for drug checking technologies

#### Patron perspectives and needs related to drug checking technologies

Consistent barriers related to drug checking technologies were reported throughout the literature by actual or potential patrons of drug checking services [9, 11–15]. These barriers include factors that directly impact patrons, such as the time required to utilize drug checking, sample quantity required for testing, the potential for sample destruction, cost, and type of results provided. In populations of people who use drugs, wait time was frequently cited as a barrier to drug checking service utilization [9, 11]. Participants from a Canadian-based qualitative study suggested that patrons would be unwilling to wait if testing their drugs pre-consumption [9]. Those entering withdrawal were seen as unlikely to utilize drug checking services, with authors reporting that a 3–6-min wait could be too long. Yet, out of the 43% of patrons who indicated a willingness to utilize drug checking services at a Canadian safe injection site (SIS), 68% would be willing to wait for up to 10 min for results [12]. Participants from another Canadian-based qualitative study suggested that maximum wait times should be limited to 30 min [13]. This sense of urgency did not appear to extend as deeply with populations of PWUPD [14]. An Australian study found that 80% of participants were willing to wait one hour for drug checking services, and the majority (61%) were willing to wait one week if it meant greater reliability of results. However, a Slovenian study that included both populations of PWUD and PWUPD found differing results [11]. About half of the PWUD indicated they would be willing to wait up to 2 months for results, while 48% of PWUPD were willing to wait up to 1 week, though no onsite services were offered. Despite indicating willingness to wait, it was also identified as a major barrier to drug checking service utilization by both groups.

Bardwell et al. described how PWUD could be a systemically vulnerable population, causing reluctance to render a drug sample for testing given the time and resources needed to obtain it [9]. This reluctance is also shown through concerns for sample destruction during testing [13]. In a US-based qualitative study of PWUD (n = 334), 94% of participants were willing to provide a drug sample for testing, which varied by quantity (residue 35%, pinhead to pinch/bump 36%, whatever it takes 24%) [15]. Meanwhile, PWUPD (n = 851) surveyed on their opinions related to drug checking services in an Australian-based study, expressed willingness to provide larger quantities relative to PWUD; scraping (98%), half of a dose (55%), and whole dose (33%) [14]. PWUPD also differ on willingness to pay for services and perceptions of cost as a barrier; 93% indicated they would pay up to $5, and 68% up to $10, while PWUD have reported that drug checking services should be free or low-cost to facilitate participation [13, 14].

People who use drugs expressed a desire for drug checking technologies that are highly accurate [9, 13]. The importance of receiving quantitative results with mixture analysis was emphasized by drug checking service users, who suggested that it could impact how they use or sell drugs [9, 16, 17]. Patrons also expressed concerns regarding detection limits (specifically, with FTIR) and the potential for false negative results with substances at low concentrations, such as potent fentanyl analogs [9]. This was corroborated by organizational stakeholders of US-based drug checking services, who expressed concerns over potential false negative results causing harm and creating liability [18].

Needs from drug checking technologies appear to differ by population. A dichotomy was shown between users of opioids and users of stimulants [19]. Patrons who use opioids desired quantitative results with mixture analysis, while users of stimulants were primarily concerned with identifying fentanyl. In Barratt et al., 53% of PWUPD indicated they would use a service that provided less than completely reliable results, while 63% reported they would use a service that did not provide completely comprehensive results [14]. Respondents indicated that comprehensive quantitative results were the most desirable (92%); however, this was closely followed by comprehensive qualitative results (89%). This suggests that identifying the presence of a substance satisfies most of the needs of this population. Moreover, in Sande et al., identification of adulterants was cited as important by PWUPD more frequently than PWUD (95% vs. 34%) [11].

#### Fentanyl test strips

Patrons expressed a desire to be able to perform drug checking at home [15, 17, 20, 21]. In a US-based qualitative study, participants showed greater interest in take-home FTS compared to onsite machines or onsite FTS (89% vs. 75.1% vs. 77.8%) [15]. FTS were consistently and highly accepted, with ≥ 90% of participants indicating that they felt confident in using FTS, that the FTS instructions were easy to follow, the results were easy to interpret, and that they would use FTS again [17, 20, 21]. A take-home FTS program found high agreement between positive results from FTS (89.9%) and onsite drug checking (89.1%) of opioid samples [20]. Results of fentanyl positivity were mixed when testing stimulant samples, with FTS indicating positive results more frequently than onsite methods (27.6% vs. 5.2% for methamphetamine; 17.2% vs. 1.1% for cocaine).

Reed et al. interviewed 29 PWUD in the USA to understand their experiences with FTS [17]. Reported barriers to FTS included lack of water needed for testing, no place to conduct the test, fear of wasting the drug being tested (specific to crack cocaine), and accessibility issues (unsure where to obtain FTS) [17]. Some participants indicated that FTS were quick to use, while other expressed concern over the time required. Additionally, a few participants indicated confusion when interpreting test results. Regarding accessibility, Krieger et al. reported potential locations for FTS distribution, which were suggested by drug checking service patrons [21]. These locations included community health clinics (57%), community-based organizations (57%), pharmacies (52%), health departments (52%), and needle exchange programs (49%).

False-positive results with FTS have been reported with methamphetamine, MDMA, and diphenhydramine (a cutting agent), with critical levels identified between 1 and 2 mg/mL [22]. Sufficient dilution of samples was recommended by the study researchers as a simple technique for preventing false-positive results, given the low limit of detection needed to identify fentanyl. Furthermore, Glick et al. reported that drug checking service stakeholders acknowledged that limitations of FTS include a lack of quantitative results and potential for user error [18]. However, stakeholders felt FTS had a place in harm reduction by helping bridge connections to PWUD because of its simplicity, ease of integration into drug checking services, and elimination of the need for PWUD to transport drug samples.

#### Fourier-transform infrared spectroscopy

The common combination of FTIR and FTS technologies is reported in the literature with mixed results [18, 23–25]. In 2018, Tupper et al. reported the successful use of FTIR in combination with FTS at a supervised consumption space (SCS) facility [23]. The drug checking service identified fentanyl in 90% of expected heroin samples and identified other dangerous substances, including “bath salts” in an expected MDMA sample, cocaine cut with a pumice stone, and plaster in an expected heroin sample. Additionally, the authors found that drug checking service utilization was higher with FTIR and FTS than in an earlier project with FTS alone (24 per 6-h shift vs. 5 per 18-h shift), with clients of the SCS reporting interest in the additional information obtained with FTIR.

Green et al. conducted a study to compare the validity of FTS, Raman spectroscopy, and FTIR against laboratory-confirmed GC–MS for the detection of fentanyl [24]. They found that FTS had the lowest limit of detection (0.1mcg/mL), highest sensitivity (96.3–100%), and comparable specificity (90.4–98.1%). Raman spectroscopy with SERS kit had variable sensitivity (38.5–61.1%), and the highest specificity (91.5–92.3%). FTIR had the poorest detection limit at 3–4% by weight, but high sensitivity (83.3%) and specificity (90.2%). The authors concluded that FTS is a suitable option if detection of the presence of fentanyl is the only desirable outcome. If more information is desired, FTS can be paired with another technology.

In a US-based qualitative study, organizational staff members of a drug checking service shared experiences using high pressure mass spectrometry (HPMS) and FTIR for drug checking, and emphasized the technical complexity of operating the machines [25]. Drug checking was initially offered with HPMS, but due to growing frustrations with its operation, it was restricted to a fixed site. An FTIR machine was found to be more appropriate for mobile outreach and obtained at a lower acquisition cost ($65 K for HPMS vs. $40 k for FTIR). Moreover, Glick et al. reported that drug checking service stakeholders were pleased with the size of FTIR and Raman machines because they were relatively small and potentially portable (TruNarc and Bruker Alpha machines) [18].

Limitations of FTIR-based combinations were also reported [26–28]. In the US-based study by Karch et al., mobile drug checking was offered to patrons, with results reported for 422 drug samples [26]. Fentanyl was detected in 134 samples using HPMS (specific for fentanyl and analogs), of which 18.7% were identified by FTIR, and 77.6% by FTS. Interpreting discordant results between the technologies was a significant challenge reported by the authors, and the lack of confirmatory testing made it difficult to compare the technologies. Additionally, a point-of-care drug checking service in Canada found that FTIR and FTS failed to identify synthetic cannabinoids in 12/25 (48%) samples confirmed positive with NMR, GC–MS or LC–MS [27]. Similarly, in another study, when benzodiazepine test strips and FTIR were used in combination for identifying novel psychoactive substances (NPS), the rate of false-positive and false-negative results was 17.8% and 29.2% [28]. Together, these point-of-care methods missed NPS in 7/113 (6.2%) of “negative” results, suggesting a need for more accurate technologies.

#### Mass spectrometry

A Canadian drug checking service interested in monitoring the local drug supply tested 2263 drug samples between 2019 and 2021 [29]. Xylazine, a veterinary anesthetic, was identified in 46 samples using GC–MS or LC–MS. Xylazine was present in 7.2% of expected opioid samples and 12.5% of expected opioid/methamphetamine samples. Newer technologies, such as portable GC–MS and paper spray mass spectrometry (PS-MS), have successfully been employed in drug checking. A Canadian drug checking service with a portable GC–MS machine identified 100% of heroin/cocaine, 95% of fentanyl, 62% of carfentanil, and 36% of etizolam-containing samples when compared against laboratory-based PS-MS testing [30]. The portable GC–MS showed inability/unreliability in detecting low concentrations of etizolam (< 3%). However, at etizolam concentrations > 3%, portable GC–MS identified 78% of etizolam-containing samples and detected lower concentrations of carfentanil (0.13–0.63%). In contrast, FTIR detected 9% of etizolam-containing samples and failed to detect carfentanil, ANPP, or heroin. When compared to FTIR, speed was a trade-off with MS technologies due to sample preparation and increased run time. Similar to other studies, the authors also emphasized the need for knowledgeable and trained technicians to run MS machines. Aside from directly acquiring MS technologies, drug checking services may address technological limitations through partnership, such as a local university owning more advanced drug checking technologies as described by Carroll et al. [25].

Borden et al. described the use of PS-MS during a pilot test in a Canadian drug checking service [31]. Using PS-MS, the authors detected fentanyl in concentrations ranging from 0.3% to the upper limit of 10%. Furthermore, etizolam was found in concentrations ranging from 0.68 to 8.27%. The median concentrations of the aforementioned substances were both below the lower detection limit of FTIR (fentanyl: 3.3%, etizolam: 2.5%). The PS-MS workflow was reported to be completed in approximately 5 min, representing potentially short wait times. The authors balance the positive performance of PS-MS with considerations on cost (“a few $100,000”), expertise for methodology development and maintenance, and physical requirements needed of the site.

### Venue

#### Patron perspectives related to location and accessibility

Concerns over the accessibility of drug checking services were frequently cited in the literature [9, 11, 13–15]. Bardwell et al. interviewed PWUD who utilized a Canadian drug checking service. PWUD felt that drug checking services should be located in close proximity to where patrons reside and congregate, and that the need to travel would be a barrier to service uptake [9]. Drug checking service location and restrictive hours of operation were also expressed as potential barriers by participants in a Slovenian-based qualitative study of PWUD and PWUPD (n = 554) [11]. Preferred locations for a drug checking service varied across studies [13, 15]. Health clinics, SSPs, treatment programs, SCSs, pharmacies, supported housing buildings, drop-in centers, medical clinics, and emergency rooms were all suggested by study participations as locations for drug checking services. Wallace et al. found that PWUD preferred services that were open 24 h per day and provided at multiple locations in order to reach different geographic areas [13]. Of 851 PWUPD surveyed in Barratt et al. most indicated that they would use a device for self-testing onsite at a festival/club (94%) or a fixed-site drug checking service (85%) [14]. Fewer indicated interest in using a mail in service (53%).

Plausible deniability, the concept that attendance to a drug checking service location could be due to reasons other than drug checking, is important to PWUD who recognize stigma associated with drug checking [32]. Mobile drug checking services could facilitate outreach to more rural participants, and patrons who avoid fixed locations due to stigma [13]. Mobile drug checking services were also supported by drug checking service stakeholders, who saw a means to provide services where PWUD reside and respond to trends in timing/location or drug use [18]. Beaulieu et al. performed a cross-sectional analysis exploring the relationship between substance type submitted for drug checking, and the timing of drug checking utilization (pre- vs. post-consumption) [33]. A stronger association was identified with pre-consumption drug testing in areas outside of a Canadian drug scene epicenter (odds ratio (OR) = 2.33; 95% confidence interval (CI) 1.51–3.56) compared to inside (OR = 1.33; 95% CI 1.09–1.63). This suggests that concern for drug adulteration may vary by region and could provide an opportunity to target services by location.

#### Integration of drug checking services

Coupling drug checking with other services could be a facilitator for patrons who already utilize harm reduction services [13]. Wallace et al. described how PWUD may have previously established trust and a sense of safety associated with these sites, which could facilitate use of drug checking services at the same location [13]. Furthermore, in a study of 180 PWUD at a Canadian SIS, 43% of people who inject drugs indicated willingness to utilize drug checking services [12]. While conducting an evaluation of FTS at a Canadian SIF, Karamouzian et al. found higher odds of overdose with positive FTS results when tested post-consumption (OR = 4.95, 95%CI 1.97–12.39), and higher odds of dose reduction following a positive FTS result (OR = 9.36, 95%CI 4.25–20.65) [34]. It is possible the counseling from SIF staff played a role in behavior modification leading to a positive impact on overdose rates. Moreover, drug checking can also facilitate an introduction or connection to other harm reduction services, such as human immunodeficiency virus (HIV) care or discussion of health-related prevention strategies [25].

Varied opinions were also expressed on drug checking service integration with other harm reduction services [19, 35]. Olding et al. reported challenges associated with offering multiple harm reduction services, including space restrictions, and effectively managing noise and protecting confidentiality for patrons [19]. During peak visit times, the authors reported that wait times for drug checking could reach one hour. Furthermore, integration of services may negatively impact PWUPD [35]. Discomfort was expressed by PWUPD in utilizing a drug checking service within an OPS (overdose prevention site), suggesting that the service was intended for dependent drug users and that resources were too constrained to serve recreational users. Moreover, as described previously, the concerns over stigma associated with drug checking service sites may act as a barrier to some patrons [32].

#### Other

Drug checking service staff and patrons have described a number of factors that play a role in the success of a service [13, 25]. Drug checking service staff members in a study by Carroll et al. reported that chemistry expertise is important, but drug checking also requires knowledge of drug effects, the local drug supply, and ideally, harm reduction experience, and personal experience [25]. Patrons also emphasized a need for skilled technicians and peers or people with lived experience who are essential to developing trust and understanding [13]. PWUD are a marginalized population subject to stigma and trauma, and a drug checking service should, consequently, be a trauma-informed service. Similarly, communications made by a drug checking service should involve a number of considerations [13]. PWUD described that the communication of drug checking results should not only include quantitative information, but also the drug/substance effects. Furthermore, drug checking service patrons represent a diverse population, and communications should remain neutral and appeal to varying levels of literacy. Betzler et al. surveyed 719 PWUPD in Germany and most agreed that the inclusion of consultation with drug checking results would be useful (79.3%) [36].

### Legality

Legality was one of the most common barriers to drug checking reported among populations of PWUD and PWUPD [13, 14, 17, 18, 25, 35, 36]. Patrons fear criminalization due to possession of drug-testing equipment and/or illegal substances, policing, surveillance, and confiscation [13, 14, 35, 36]. A US-based drug checking service experienced ~ 50% decline in visits (from an average of 42 visits per month to 23 visits per month) after a local initiative led to increased policing [25]. Drug checking service staff described how patterns of police violence impacted PWUD and led to difficulties engaging with them. Concerns over the legality of drug checking varied by location. In one particularly high-crime area, many patrons felt that police had more pressing matters to attend to, and they should not have concerns carrying FTS if they are legal [17]. Authors in 2 studies of drug checking service stakeholders and patrons, respectively, concluded that establishing supportive policy and relationships with law enforcement were seen as a potential facilitator to drug checking service utilization [13, 17]. Moreover, of 851 PWUPD surveyed in Barratt et al. 97% would use a drug checking service if police showed support by keeping clear [14].

Legal implications extend beyond patrons [25, 37]. Drug checking service organization staff interviewed in Carroll et al. became uncomfortable offering drug checking at mobile sites after learning that testing trace substances would constitute probable cause for arrest [25]. This was especially salient for staff members with a criminal record. The “gray area” of legality also led to apprehension in discussing drug checking with partnering clinicians. Similarly, in a population of PWUPD, legality of home drug-testing kits was seen as a major barrier given concern that test kits would link them to possession charges [37]. Many participants cited the “RAVE” act (subsequently renamed the Illicit Drug Anti-Proliferation Act) as a cause for event/festival leadership to deny event admission with test kits due to fear of liability and being viewed as condoning drug use on the premise.

### Privacy

Confidentiality and anonymity were identified as important features of a drug checking service [11, 13]. Patrons expressed concern for identification through their physical attendance at a drug checking site. Additionally, the inappropriate use of data and data sharing was another mechanism of concern. While benefits of drug checking integration were previously discussed, coupling with other services was also seen as a barrier to some who fear being identified or surveilled [13].

Beyond patrons of drug checking services, privacy was also a concern for drug dealers [38]. Not only could they be exposing themselves to criminalization but they could compromise their reputation by showing low confidence in their products through testing. Home drug-testing kits were suggested to support anonymity. Privacy also extends to the distribution of drug checking results, as described by Barratt et al. [14]. The vast majority of PWUPD included in the study indicated that they would use a drug checking service that provided individual, confidential results (97%) or individual and deidentified public results (95%). Fewer participants indicated willingness to receive results through a public channel, such as a website (36%).

## Discussion

Patrons to drug checking services described many personal considerations that impact service utilization. Drug checking technologies should be highly accurate and minimize the risk associated with newer, high-potency drugs. The desire for quantitative results was seen throughout populations of PWUD, who appeared more concerned with the amount of adulterant (e.g., % fentanyl) present in a sample, than its presence alone (e.g., fentanyl present/not present). Both FTIR and MS offer desirable, quantitative results and have been employed in harm reductions settings. Potential considerations for MS, compared to FTIR, include a trade-off between improved technological specifications, and the increased cost, decreased portability, greater expertise required for operation, and destructive nature of the testing.

The goals of a drug checking service may also dictate the technological requirements. In order for drug checking services to identify new substances and provide confirmatory testing, more advanced drug checking technologies must be employed (such as MS technologies). The literature showed examples of implementation of these drug checking technologies at fixed sites, mobile sites, and through partnerships (e.g., university or other professional laboratories). Moreover, drug checking technologies are not a one-size-fits-all solution and should be considered on an individualized basis within the context of the region, evolving drug markets and goals of the harm reduction service. For example, while FTS may be a fundamental technology for US-based services, their utility may be limited in regions less impacted by opioids, or in areas with analogs not detected by FTS.

The drug checking technologies required to meet the needs of PWUD and PWUPD could be a target for implementing harm reduction services in different settings (e.g., the choice of drug checking technology at an OPS vs. a festival). Other considerations include location, mobility of services, and integration of drug checking with other services. These represent actionable service components which may improve service utilization by increasing accessibility. Furthermore, drug checking service patrons emphasized the need for anonymity and trust. Representative staff, such as peers and people with lived experience, may help build trust.

The literature outlined how a drug checking service can be influenced by the population served and the local climate for drug checking. For instance, Carroll et al. described how drastically local policing initiatives impacted drug checking service utilization. Moreover, the population of patrons served can differ in many ways, such as the types of drugs used, patterns of use, willingness to utilize drug checking, and impact of drug checking on behavior [4]. This highlights the need to obtain information specific to the implementation site as feasibility and barriers can vary between individual harm reduction agencies.

### Gaps in the evidence

We identified several literature gaps that include challenges related to drug checking policy, lack of information regarding data sharing practices for drug checking, and lack of established methodology for evaluation of drug checking services. Patrons frequently cited legality as a barrier to drug checking service utilization. Anonymity and protection from criminalization are necessary to build trust and facilitate comfort with drug checking services. Some literature exists on the legal environment for drug checking in the USA, such as Davis et al. [39]. However, summarizing the literature on the legal environment is difficult due to the ever changing nature of policy.

To our knowledge, no comprehensive literature has been published on the privacy of drug checking services, despite the recognition of this factor as a barrier to the implementation of drug checking technologies [11, 13]. Data sharing by drug checking services remains a concern among patrons who fear a breach of confidentiality. However, if drug market monitoring and subsequent harm reduction messaging are to be employed, data sharing must occur in some capacity. The extent of data sharing, how it is shared, with whom it is shared, and considerations for data sharing agreements have not been reported on in the literature but remain important considerations.

Lastly, the methodology for evaluating drug checking services has not been explored in populations of PWUD. As described by Wallace et al., PWUD believe that disposal of drugs is an unrealistic expectation and that the valuation of a drug checking service should rely on other outcomes, contrary to prior research in populations of PWUPD [13]. Budgetary constraints and the need for impactful, measurable public health initiatives could create pressure on drug checking services to showcase their value. The value of drug checking services should be further explored in this context to better identify meaningful and quantifiable outcomes for harm reduction. Furthermore, while some research has investigated the communication of results to patrons [13], the optimal method has not yet been elucidated and could provide an important avenue for future research.

### Limitations of review

Given the nature of this scoping review, the results do not provide an in-depth evaluation of drug checking or assess biases present in the literature. This review is intended to be exploratory and guide future discussions with drug checking stakeholders. Furthermore, this review does not focus on the technical aspects associated with various drug checking technologies. While highly informative, reviews of this nature have been published previously [7].

## Conclusion

Our goal was to identify contextual factors impacting drug checking services to aid in decision-making on the choice of technology and service implementation. The twenty-nine studies included in this scoping review were diverse, with varying methods, populations, services, and locations. This diversity highlights the need to understand local populations and climates to successfully implement a drug checking service. Overall, common themes emerged regarding drug checking technologies, venue, legality, and privacy associated with drug checking. We intend to utilize these points to guide future discussions with local drug checking stakeholders.

### Supplementary Information

**Additional file 1:** Included studies and key findings.

## Data Availability

Not applicable.

## Abbreviations

CDC: Centers for disease control and prevention
FTIR: Fourier-transform infrared spectroscopy
FTS: Fentanyl test strip
GS-MS: Gas chromatography with mass spectrometry
HIV: Human immunodeficiency virus
HPMS: High pressure mass spectrometry
LC-MS: Liquid chromatography with mass spectrometry
MS: Mass spectrometry
NPS: Novel psychoactive substances
OD2A: Overdose Data to Action
OPS: Overdose prevention site
PS-MS: Paper spray mass spectrometry
PWUD: People who use drugs
PWUPD: People who use party drugs
SCS: Supervised consumption space
SIS: Safe injection site
