# Supplementary material for: Contextual factors affecting the implementation of drug checking for harm reduction: a scoping literature review from a North American perspective
Source: Harm Reduct J. 2023 Sep 4;20:124. doi: 10.1186/s12954-023-00856-0 (PMC10478363; doi:10.1186/s12954-023-00856-0)
Supplement: Supplementary file 1 — Additional file 1: Included studies and key findings. [file 12954_2023_856_MOESM1_ESM.docx]

**Table 2**. *Studies Related to Drug Checking Services and Technologies*^a^

| **First Author (Pub. Year)** | **Study design** | **Country** | **Population: Study Participants** | **Technologies Described** | **Area of Outcomes** | **Main Findings** |
| --- | --- | --- | --- | --- | --- | --- |
| Lockwood 2021 | Diagnostic test accuracy study (n=N/A) | United States | N/A | FTS | *Technology*: FTS | - False positive results with FTS were reported with methamphetamine, MDMA, and diphenhydramine (cutting agent), with critical levels between 1-2mg/mL.  Sufficient dilution of a sample needed to combat false positive results. |
| Karch 2021 | Diagnostic test accuracy study (n=422 samples) | United States | Use Disorder | FTS, FTIR, HPMS | *Technology*: FTIR Combinations | - HP-MS detected fentanyl in 134 samples; of which 18.7% were identified by FTIR, and 77.6% by FTS. - Several samples (n=19) were not able to be processed by FTIR due to quantity or form. - Discordant results between the technologies were a major challenge, particularly between FTS and HP-MS. |
| Green 2020 | Diagnostic test accuracy study (n=210 samples) | United States | Mixed | FTS, FTIR, Raman spectrometry,  Confirmatory Testing (GC-MS) | *Technology*: FTIR Combinations | - FTS had the lowest limit of detection (0.1mcg/mL), highest sensitivity (96%, 100%), and comparable specificity (90.4%, 98.1%). - Raman spectroscopy with SERS kit had variable sensitivity (38.5%, 61.1%), and the highest specificity (91.5%, 92.3%). - FTIR had the highest detection limit at 3-4% by weight, and high sensitivity and specificity (83.3%, 90.2%). - If qualitative results for fentanyl are desired, FTS should be used. If additional information is desired, FTS should be paired with another technology. |
| Laing 2021 | Diagnostic test accuracy study (n=159 samples) | Canada | Use Disorder | FTIR, BZD test strips,  Confirmatory Testing (GC-MS, LC-MS, NMR) | *Technology*: FTIR Combinations | - When BZD test strips and FTIR results were taken in consort for the detection of NPS, the rates of false positives and false negatives were 17.8% and 29.2%. - Together these point-of-care methods missed NPS in 6.2% (7/113) of samples, which suggests a need for more robust technologies for NPS monitoring. |
| Ti 2021 | Diagnostic test accuracy study (n=440 samples) | Canada |  | FTIR, FTS,  Confirmatory Testing (NMR, GC-MS, LC-MS) | *Technology*: FTIR Combinations | - Point of care testing with FTIR and FTS failed to identify synthetic cannabinoids in 12/25 (48%) samples confirmed by NMR, GC-MS, LC-MS. |
| Tupper 2018 | Prevalence study (n=1714 samples) | Canada | Use Disorder | FTIR, FTS | *Technology*: FTIR Combinations | - The authors report successful use of FTIR in combination with FTS in a point of care setting (a SCS). - Of 907 expected heroin samples, only 17.6% contained heroin while 90% contained fentanyl. Other dangerous substances were also identified including "bath salts", pumice stone, and plaster. - The utilization of FTIR with FTS was higher than an earlier project with FTS alone. |
| Borden 2022 | Diagnostic test accuracy study (n=113 samples) | Canada | Use Disorder | PSMS | *Technology*: Mass Spectrometry | - PS-MS detected etizolam in 10 samples (9% of all samples and 17% of expected opioid samples).  PS-MS detected fentanyl ranging from 0.3% to the upper limit of 10%, and etizolam in concentrations ranging from 0.68% to 8.27%. The median concentrations were below the lower detection limit of FTIR. - The PS-MS workflow was ~5 minutes. - Barriers of PS-MS include cost ("a few $100,000"), expertise for methodology development and maintenance, and physical requirements of the site. |
| Bowles 2021 | Diagnostic test accuracy study (n=2263 samples) | Canada | Mixed | GC-MS, LC-HRMS | *Technology*: Mass Spectrometry | - Xylazine, a veterinary anesthetic, was identified in 46 samples using GC-MS and/or LC-MS. This represents 7.2% of expected opioid samples, and 12.5% of expected opioid/methamphetamine samples. |
| Gozdzialski 2021 | Diagnostic test accuracy study (n=59 samples) | Canada | Mixed | Portable GC-MS, FTIR  Confirmatory Testing (PSMS) | *Technology*: Mass Spectrometry | - Portable GC-MS identified 100% of heroin or cocaine, 95% of fentanyl, 62% of carfentanil, and 36% of etizolam containing samples. - FTIR detected 9% etizolam containing samples, and failed to detect carfentanil, ANPP, and heroin. - Portable GC-MS is slower than FTIR due to sample preparation and increased run time. Requires knowledgeable and trained technicians. |
| Reed 2022 | Qualitative research: Interviews (n=29) | United States | Use Disorder | FTS | *Technology*: Patron Perspectives on FTS  *Legality* of drug checking | - FTS reported as easy to use. - Main barriers included fear of wasting drug, lack of access, and not having a location to use FTS. - Some desired additional quantitative results/mixture analysis. - Concerns over the legality of FTS varied. |
| Bardwell 2019 | Qualitative research: Interviews (n=20) | Canada | Use Disorder | FTIR, FTS, Colorimetric, TLC, Capillary electrophoresis UV, HPLC-UV, HPLC-MS | *Technology*: Patron Perspectives related to drug checking technologies  *Venue*: Patron Perspectives | - Barriers to drug checking included sample destruction, wait time, and location (if travel is required). - Quantitative results were viewed as important. Concerns expressed over FTIR limits of detection. - drug checking service should be located close to where patrons reside or congregate. |
| Klaire 2022 | Cross sectional study (n=218 participants) | Canada | Use Disorder | FTS | *Technology*: Patron Perspectives on FTS | - ~95% indicated that FTS instructions were easy to follow, results were easy to interpret, and intention to use FTS again. - High agreement found between positive fentanyl results from take-home FTS (89.9%) and onsite drug checking (89.1%) of opioid samples. Results of fentanyl positivity were mixed for stimulant samples. |
| Kennedy 2018 | Cross sectional study (n=180 participants) | Canada | Use Disorder | N/A | *Technology*: Patron Perspectives related to drug checking technologies  *Venue*: Integration of Services | - Of patrons who indicated willingness to utilize drug checking service at an SIS (43%), 68% would be willing to wait for up to 10 minutes for results. - 43% of PWID indicated willingness to utilize drug checking services within an SIS. |
| Carroll 2022 | Qualitative research: Interviews (n=12) | United States | Use Disorder | HPMS, FTIR | *Technology*: Patron Perspectives related to drug checking technologies  *Venue*: Integration of Services  *Legality* of drug checking  *Other* (Staff Experience) | - An FTIR machine was found to be more appropriate for mobile outreach than HPMS. Reported acquisition costs of $65K for HPMS, and $40k for FTIR. - Partnership with a local university facilitated confirmatory testing. - drug checking service offered onsite at an SSP, and at outreach collaborations. drug checking helped facilitate connection to other services (e.g., HIV care). - Legality identified as a major barrier, causing discomfort with mobile drug checking and apprehension to discuss drug checking with partnering clinicians. - Increased policing led to a major decrease (~50%) in drug checking service utilization. - Drug checking requires expertise in chemistry, knowledge of drug effects, the local drug supply, and ideally, harm reduction and/or personal experience. |
| Sherman 2019 | Qualitative research: Survey (n=334) | United States | Use Disorder | FTS, "on site machines" | *Technology*: Patron Perspectives related to drug checking technologies, and Perspectives on FTS  *Venue*: Integration of Services | - >85% of participants desired quantitative results - 94% of participants were willing to provide a sample for testing (residue 35%, pinhead to pinch/bump 36%, whatever it takes 24%) - Greater interest in take-home FTS compared to onsite machines or onsite FTS (89% vs. 75.1% vs. 77.8%). - Half of participants expressed interest in using drug checking services daily. - Preferred location varied. The most frequently mentioned included SSPs, health clinics, treatment programs, and safe consumption sites. |
| Wallace 2020 | Qualitative research: Interviews (n=27) | Canada | Use Disorder | N/A | *Technology*: Patron Perspectives related to drug checking technologies, and Perspectives on FTS  *Venue*: Paton Perspectives, Integration of Services  *Legality* of drug checking  *Privacy* Concerns Related to drug checking service  *Other* (Staff Experience) | - Participants desired quantitative results with high accuracy. - Barriers include cost, wait time (30min max), sample destruction, and fears of criminalization, surveillance, and inappropriate data sharing. - Participants expressed desire to perform testing at home. - Drug checking service should be provided at multiple locations, and ideally offered 24/7. Mobile drug checking services suggested to reach rural patrons, or those who fear stigma of fixed sites. Pharmacies, supported housing buildings, drop-in centers, medical clinics and emergency rooms were suggested as potential drug checking service locations. - Coupling drug checking with other services was seen as a facilitator for patrons who already utilize harm reduction services, and a barrier to some who fear being identified or surveilled. Suggested sites for integration included OPS and SIS. - Results should include quantitative information, and drug effects. Language should be neutral and appeal to varying levels of literacy. - Staff should include skilled technicians, and harm reduction workers. Peers or people with lived experience are needed to develop trust and understanding. - Drug checking service should be a trauma-informed service. |
| Krieger 2018 | Qualitative research: Survey (n=81) | United States | Mixed | FTS | *Technology*: Patron Perspectives on FTS | - >95% felt confident in using FTS and indicated desire to use FTS in the future. - Participants felt comfortable obtaining FTS from a variety of locations; community health clinics (57%), community-based organizations (57%), pharmacies (52%), health departments (52%), and needle exchange programs (49%). |
| Goodyear 2020 | Qualitative research: Interviews (n=50) | Canada | Mixed | N/A | *Venue*: Patron Perspectives  *Legality* of drug checking | - Integration with OPS may discourage PWUPD from utilizing drug checking service. - Barriers included fear of confiscation and criminalization due to possession. |
| Bardwell 2019 | Qualitative research: Interviews (n=20) | Canada | Use Disorder | N/A | *Privacy* Related to drug checking service | - Potential for positive trickle-down effect from dealer drug checking service utilization. - Privacy is a barrier for drug checking service utilization by drug dealers, fearing criminalization and being seen as having a lack of confidence in their product. - Home testing kits suggested to support anonymity. |
| Measham 2020 | Mixed methods (n=171 samples, n=144 consultations) | UK | Mixed | FTIR, FTS, Colorimetric, UV, Mass loss analysis, ASAP-MS | *Venue*: Patron Perspectives | - Stigma reported as a barrier to utilizing drug checking service apart of drugs services. - Plausible deniability associated with a location is a facilitator. |
| Olding 2020 | Qualitative research: Interviews (n=91) | Canada | Use Disorder | FTS, FTIR | *Technology*: Patron Perspectives related to drug checking technologies  *Venue*: Integration of Services | - Patrons who use opioids desired quantitative results with mixture analysis, while users of stimulants were primarily concerned with qualitative identification of fentanyl. - Challenges associated with offering multiple harm reduction services included space restrictions, effectively managing noise, and protecting patron confidentiality. |
| Palamar 2019 | Qualitative research: Interviews (n=32) | United States | PWUPD | N/A | *Legality* of drug checking | - Legality identified as a major barrier to home-test kit use. Concerns included cause for possession charges, and denial of entry at events/festivals due to fear of liability from administration. |
| Karamouzian 2018 | Cross sectional study (n=1141 samples) | Canada | Use Disorder | FTS | *Venue*: Integration of Services | - The SIF found 1% utilization of onsite FTS, with 58% of checks performed post-consumption.  Significantly higher odds of overdose were found for positive results when tested post-consumption (OR=4.95, 95%CI: 1.97-12.39), but not pre-consumption. For patrons who tested pre-consumption, there was a 9.36 times higher odds of dose reduction following a positive result (95%CI: 4.25-20.65). - Counseling from SIF staff may have had a positive impact on rates of overdose. |
| Sande 2018 | Qualitative research: Survey (n=554) | Slovenia | Mixed | N/A | *Technology*: Patron Perspectives related to drug checking technologies  *Venue*: Patron Perspectives  *Privacy* Concerns Related to drug checking service | - Barriers included wait times, inability to collect samples in the field, fear of loss of anonymity, and accessibility (location and hours of operation). - About half of the high-risk participants indicated they would be willing to wait up to 2 months for results, while 48% of PWUPD were willing to wait up to 1 week.  13% of the participants who use party drugs indicated that they would be willing to pay for faster results. [No onsite testing was offered] - Fixed drop-off points for samples was seen as a barrier by PWUPD in small/rural communities, given that anonymity could be compromised. |
| Betsos 2021 | Qualitative research: Interviews (n=26) | Canada | Use Disorder | N/A | *Technology*: Patron Perspectives related to drug checking technologies | - Drug dealers indicated desire for quantitative results and mixture analysis. - Concerns expressed over fentanyl analogs and need for technologies with a lower detection limit than FTIR. |
| Glick 2019 | Qualitative research: Interviews (n=32) | United States | Organizational stakeholders | FTS, FTIR, Raman spectrometry | *Technology*: Patron Perspectives on FTS  *Venue*: Patron Perspectives  *Legality* of drug checking | - Stakeholders desired highly accurate technologies and were most concerned with false negative results causing harm and liability. - Despite lack of quantitative results and potential user error, FTS have a place in harm reduction by bridging connections to PWUD, ease of use, ease of integration into drug checking service, and eliminating the need sample transport. - Respondents were pleased with the size and portability of machines (TruNarc and Bruker Alpha). - Mobile drug checking service were supported by many stakeholders. - Legality of drug checking service was of major concern; emphasized need for supportive policy and relationships with law enforcement. |
| Beaulieu 2021 | Cross sectional study (n=3561 samples) | Canada | Use Disorder | FTIR, FTS | *Venue*: Patron Perspectives | - Concern for drug adulteration might vary by region and could provide an opportunity to target drug checking service by location. - There was a stronger association with pre-consumption drug testing in areas outside of the drug scene epicenter (ORMH=2.33; 95% CI 1.51-3.56) compared to inside (ORMH=1.33; 95% CI 1.09-1.63). |
| Barratt 2018 | Qualitative research: Survey (n=851) | Australia | PWUPD | N/A | *Technology*: Patron Perspectives related to drug checking technologies  *Venue*: Patron Perspectives  *Legality* of drug checking  *Privacy* Concerns Related to drug checking service | - Most participants were willing to wait one hour, provide ½ dose for testing, and pay for drug checking service. - 53% of participants indicated they would use a service that provided less than completely reliable results, or a service that did not provide completely comprehensive results (63%). - Respondents indicated that comprehensive quantitative results were the most desirable (92%), followed by comprehensive qualitative results (89%). - Most participants indicated interest in self-testing, using drug checking service onsite at a festival/club, and fixed-site drug checking service (85%). Fewer indicated interest in using a mail-in service (53%). - Criminalization was identified as a major barrier. 94% would not use a service if arrest was possible. - Most participants would use a service that provided individual, confidential results (97%) or individual and deidentified public results (95%). Fewer indicated willingness to receive results through a public channel, such as a website (36%). |
| Betzler 2021 | Qualitative research: Survey (n=719) | Germany | PWUPD | N/A | *Legality* of drug checking  *Other* (Consultations) | - Criminal prosecution (40.1% indicated total agreement), and concerns for privacy (27.1% indicated total agreement) were the major barrier identified. The risk of being seen by others, and concern for time to use drug checking service, were not major concerns. - 79.3% of participants indicated that consultation could be useful to patrons. |

^a^ ANPP = 4-anilino-N-phenethylpiperidine, BZD = benzodiazepine, FTS = fentanyl test strips, FTIR = fourier transform infrared spectroscopy, GC-MS = gas chromatography mass spectrometry, HPMS = high pressure mass spectrometry, HPLC-MS = high pressure liquid chromatography with mass spectrometry, HPLC-UV = high pressure liquid chromatography with ultra-violet, HPMS = high pressure mass spectrometry, LC-MS = liquid chromatography mass spectrometry, LC-HRMS = chromatography high resolution mass spectrometry, NMR = nuclear magnetic resonance, PS-MS = paper spray mass spectrometry, PWUPD = people who use party drugs, SCS = supervised consumption spaces, SIF = safe injection facility, SIS = safe injection site, SSP = syringe service program, TLC = thin layer chromatography, UV = ultra-violet
